# Spectrotemporal Modulation Detection and Speech Perception by Cochlear Implant Users

**DOI:** 10.1371/journal.pone.0140920

**Published:** 2015-10-20

**Authors:** Jong Ho Won, Il Joon Moon, Sunhwa Jin, Heesung Park, Jihwan Woo, Yang-Sun Cho, Won-Ho Chung, Sung Hwa Hong

**Affiliations:** 1 Division of Ophthalmic and Ear, Nose and Throat Devices, Office of Device Evaluation, Center for Devices and Radiological Health, US Food and Drug Administration, Silver Spring, Maryland, United States of America; 2 Department of Otorhinolaryngology-Head and Neck Surgery, Samsung Medical Center, Sungkyunkwan University School of Medicine, Seoul, 135-710, Republic of Korea; 3 School of Electrical Engineering, Biomedical Engineering, University of Ulsan, Ulsan 680-749, Republic of Korea; Birkbeck College, UNITED KINGDOM

## Abstract

Spectrotemporal modulation (STM) detection performance was examined for cochlear implant (CI) users. The test involved discriminating between an unmodulated steady noise and a modulated stimulus. The modulated stimulus presents frequency modulation patterns that change in frequency over time. In order to examine STM detection performance for different modulation conditions, two different temporal modulation rates (5 and 10 Hz) and three different spectral modulation densities (0.5, 1.0, and 2.0 cycles/octave) were employed, producing a total 6 different STM stimulus conditions. In order to explore how electric hearing constrains STM sensitivity for CI users differently from acoustic hearing, normal-hearing (NH) and hearing-impaired (HI) listeners were also tested on the same tasks. STM detection performance was best in NH subjects, followed by HI subjects. On average, CI subjects showed poorest performance, but some CI subjects showed high levels of STM detection performance that was comparable to acoustic hearing. Significant correlations were found between STM detection performance and speech identification performance in quiet and in noise. In order to understand the relative contribution of spectral and temporal modulation cues to speech perception abilities for CI users, spectral and temporal modulation detection was performed separately and related to STM detection and speech perception performance. The results suggest that that *slow spectral modulation* rather than slow temporal modulation may be important for determining speech perception capabilities for CI users. Lastly, test–retest reliability for STM detection was good with no learning. The present study demonstrates that STM detection may be a useful tool to evaluate the ability of CI sound processing strategies to deliver clinically pertinent acoustic modulation information.

## Introduction

Speech identification performance for cochlear implant (CI) users has been gradually improving over the past 20 years [1, 2] partly due to the advancement in CI coding strategy, front-end signal processing, electrode design, and the use of electro-acoustic stimulation. However, most clinically available CI coding strategies are still variations of continuous interleaved sampling (CIS) strategy. The basic concept of CIS strategy is that the auditory-nerve fibers are stimulated by narrowband amplitude modulation (AM) of a biphasic pulsatile carrier with a constant stimulation rate through 12 to 22 implant channels. While patients with modern CI devices often show remarkable speech identification in quiet, speech perception outcomes deteriorate substantially in competing background noise.

In order to understand potential factors responsible for variability in speech identification performance across CI users, psychoacoustic tests using either spectrally or temporally modulated stimuli as a test signal have been often used. For example, spectral modulation sensitivity has been documented on spectral-ripple discrimination or spectral-ripple detection tasks for CI users. In these tests, listeners are presented with one interval containing a spectrally modulated stimulus (i.e., test signal or “oddball”) and two other intervals containing a reference signal. The spectrum of the oddball stimulus is sinusoidally modulated with a predefined modulation depth and density that varies adaptively based on the listener’s response. For the spectral-ripple discrimination test, spectral modulation depth is fixed, and spectral-ripple density thresholds are measured using a spectral phase-reversed reference signal [3–8]. In contrast, spectral modulation frequency is fixed for the spectral ripple detection test, and thresholds are measured by determining the minimum spectral modulation depth required to discriminate a noise carrier with flat spectrum from that with a sinusoidally modulated spectrum [9–11]. Significant correlations were reported between spectral modulation sensitivity (measured using both techniques) and speech perception outcomes for CI users [3, 5, 7, 11, 12].

Previous studies have also documented that temporal modulation cues are critical for speech perception outcomes in CI users. As indicated above, the design of CIS coding strategy and its variants provide limited temporal modulation information through a few spectral channels. The extent to which CI users receive temporal modulation information depends on the CI coding strategy or programming parameters such as electrode configuration, bandwidth of the channels, stimulation rates [13–15], the auditory-nerve fiber’s capacity to follow electrical temporal modulations [16–19], or different biological conditions of the implanted cochlea [20, 21]. In the temporal modulation detection test, temporal modulation frequency is fixed, and detection thresholds are measured by determining the minimum temporal modulation depth required to discriminate a unmodulated noise carrier from that with a sinusoidally modulated amplitude [16, 18, 22–26]. Previous studies have shown that CI users show quite good temporal modulation detection performance for low modulation frequencies [18, 23, 24, 26]. Furthermore, significant correlations have been reported between speech perception performance and temporal modulation detection performance measured either through sound processor [24, 26] or direct stimulation in CI users [16, 23].

**Fig 1:**
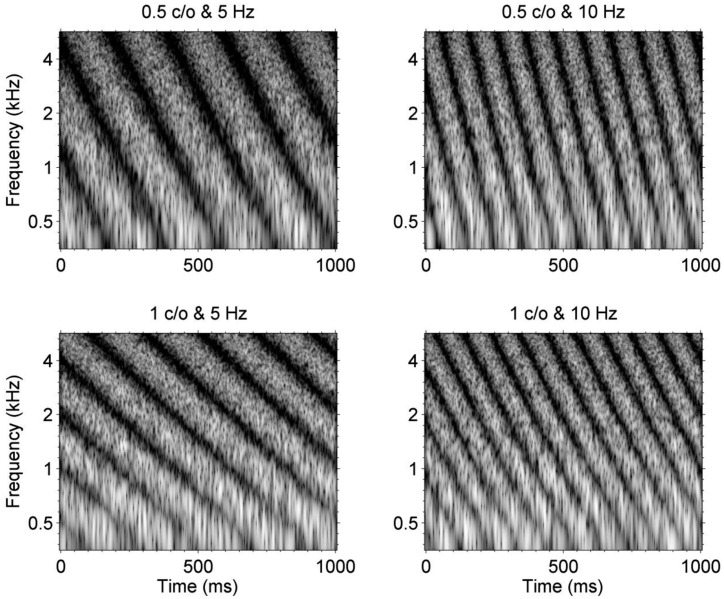
Example spectrograms for STM stimuli for four different combinations of spectral density and temporal rate. In these spectrograms, different amplitudes of the STM stimuli are depicted as a grayscale over a dynamic range of 40 dB.

These previous studies have often focused on measuring spectral or temporal modulation sensitivities *separately* and relate them to speech perception performance for CI users. Such approach may be useful to assess the contribution of a specific acoustic cue to speech perception outcomes, while controlling for possible confounding acoustic cues. However, speech is composed of dynamic spectral and temporal modulations that change over time depending on the speech utterance. It is possible that the extent to which *the combination of spectral and temporal modulation cues* is transmitted to listeners via electric stimulations through CIs may be substantially different than the extent to which such modulation cues are delivered to NH or HI listeners. To test this idea, in the current study, we employed stimuli that were originally developed to establish a model of speech perception for NH listeners based on the spectral and temporal modulation patterns of speech signals [27, 28]. These stimuli, often called “moving ripple” or “spectrotemporal modulation (STM)” stimuli, represent STM patterns that vary across frequency channels and over time. Fig 1 shows example spectrograms of STM stimuli with different combinations of spectral density and temporal rate. In the upper and lower rows, spectrograms for STM stimuli with a spectral density of 0.5 and 1.0 cycle per octave (c/o) are shown, respectively. For example, in the upper row, a relatively broad spectral modulation pattern is shown along the frequency domain. In the left and right columns, spectrograms for STM stimuli with a temporal rate of 5 and 10 Hz are shown, respectively. Here, the temporal rate determines the speed of frequency sweep that falls (i.e., from high to low frequency) along the frequency domain and repeat the frequency sweep over time. In Fig 1, a downward direction of frequency sweep is represented, but one can set up an upward direction of frequency sweep to configure STM stimuli.

**Fig 2:**
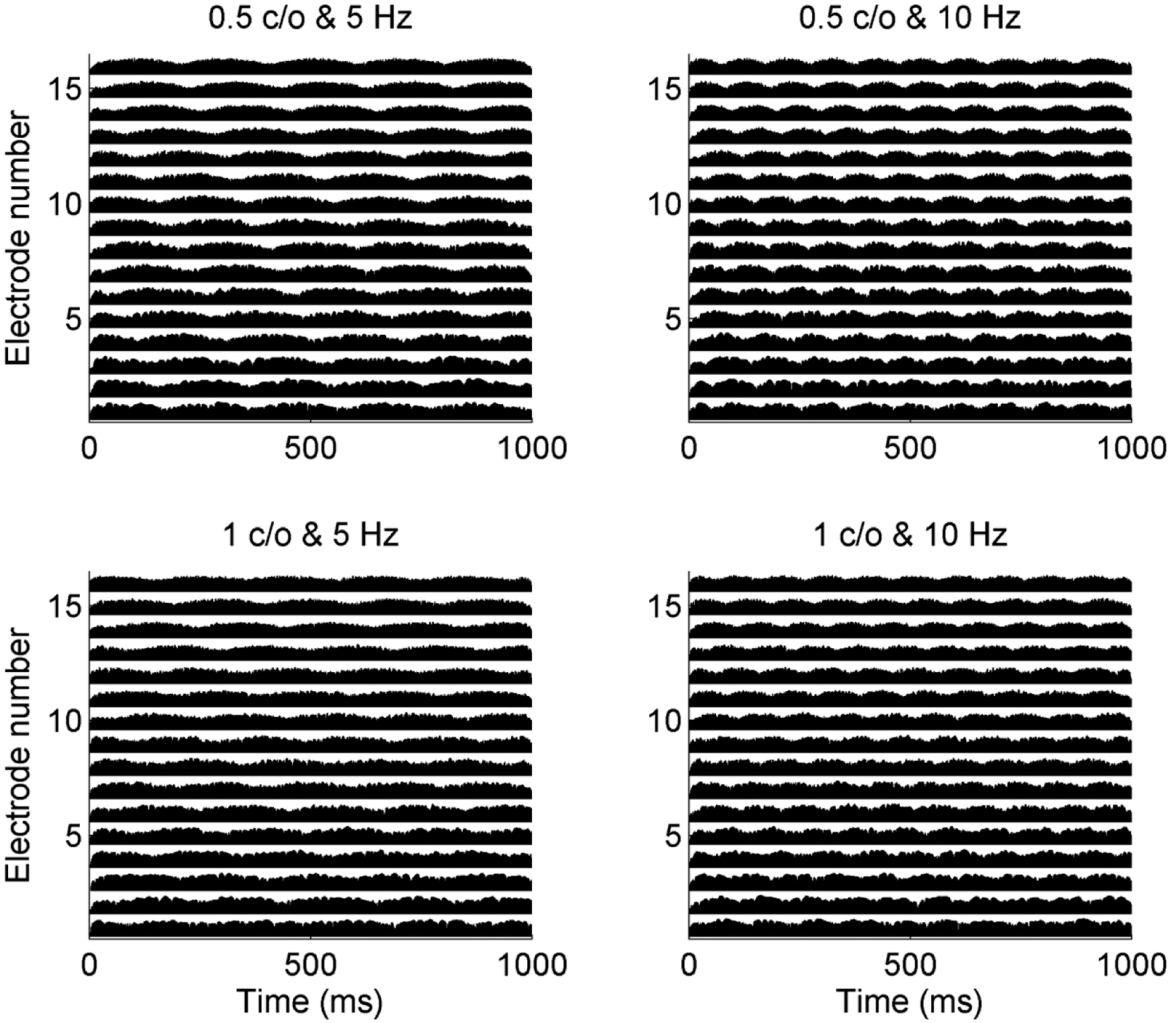
The electrode outputs in response to the four STM stimuli shown in Fig 1. For these simulations, HiResolution^®^ strategy (Advanced Bionics Corporation) was used.

Fig 2 shows how CI sound processor encodes STM stimuli. For these electrode outputs (i.e., electrodograms), STM stimuli that are shown in Fig 1 were used. HiResolution^TM^ sound processing strategy (Advanced Bionics Corporation) was used for these examples because among 23 CI subjects in the current study, 10 subjects were fitted with HiResolution^TM^ strategy. Here, higher electrode numbers indicate high frequency channels. First, compare the electrode outputs for a temporal rate of 5 and 10 Hz. For 5 Hz stimuli, each electrode represents five periods of within-channel temporal modulations, whereas for 10 Hz stimuli, ten periods of within-channel temporal modulations are shown. Next, consider temporal modulation patterns *across* electrodes. For the STM stimulus with a 0.5 c/o and 5 Hz rate, the peak of envelope modulation occurs first in the high frequency channel, because the acoustic waveform represents the high frequency component first. Subsequently, the peaks of envelope modulations for lower frequency channels occur later, and this “frequency sweep” pattern repeats at a rate of 5 Hz over time. As a temporal rate increased, for example, from 5 to 10 Hz shown in Figs 1 and 2, the speed of the frequency sweep increased. As spectral density increased from 0.5 to 1 c/o, more dense spectral modulation patterns were presented in the electrode outputs at any given time. By measuring a listener’s just-noticeable-difference for spectral modulation depth between a STM stimulus and stead-state unmodulated stimulus, CI users’ STM detection performance can be measured.

Using STM stimuli, Chi et al. (1999) [27] initially measured STM detection thresholds for normal-hearing (NH) listeners. Bernstein et al. (2013) [29] has recently measured STM detection thresholds for NH and hearing-impaired (HI) listeners to test a hypothesis that the effect of sensorineural hearing loss would be greatest at higher spectral densities because of the degraded frequency selectivity in HI listeners. This hypothesis was supported by the finding that HI listeners showed poorer STM sensitivity for higher spectral densities but not for lower spectral densities. Bernstein et al. [29] also showed a significant correlation between STM thresholds and sentence recognition in background noise for HI listeners, suggesting that altered encoding of STM cues due to sensorineural hearing loss negatively affects speech identification performance in HI listeners. Mehraei et al. (2014) [30] also examined the effects of hearing loss on STM detection thresholds in HI listeners as a function of carrier frequencies and the relationship among narrowband STM detection performance, FM detection thresholds, bandwidths of auditory filters, and sentence identification in noise. Consistent with Bernstein et al. [29], the results of Mehraei et al. [30] further supported the idea that degraded speech perception performance in HI listeners is related to poorer FM detection performance at low frequencies and reduced frequency selectivity at high frequencies.

The primary goal of the current study was to evaluate STM detection as a measure of both spectral and temporal modulation processing abilities in CI users. In addition, test-retest reliability was evaluated for the STM detection test. If the STM detection test is reliable with limited learning effects and shows predictive power for speech identification in quiet or noise for a group of CI subjects, the test could be useful as a clinical tool to evaluate the performance of individual CI users with different sound encoding strategies or programming parameters. Thus, the tests are repeated on separate days for a subset of CI subjects. Another goal of the current study was to evaluate the extent to which the STM detection test is predictive of clinically meaningful performance which may depend on STM encoding. To test this, sentence identification in quiet using the Korean Central Institute for the Deaf Sentence test (K-CID) [31], and sentence identification in background noise using Korean Hearing in Noise Test Sentence (K-HINT) [32]. As described above, the STM stimuli present *both* spectral and temporal modulation information, thereby providing a measure of combined spectrotemporal modulation sensitivity. To further understand the nature of the STM detection performance for CI users, it is important to evaluate if STM detection thresholds correlate with either spectral or temporal modulation detection thresholds. For this purpose, we also measured spectral modulation detection [9, 11, 12, 33] and temporal modulation detection [22, 26] in the same group of subjects.

Furthermore, the current study administered the aforementioned psychoacoustic tasks for NH and HI listeners. The three subject groups were expected to provide a wide range of psychoacoustic capabilities to test our primary hypothesis that altered encoding of STM information due to the sensorineural hearing loss (i.e., HI group) and the CI-auditory nerve interface (i.e., CI group) may constrain STM sensitivity *differently* from normal-hearing (i.e., NH group). Previous studies (e.g., [34–35]) have demonstrated that CI users behave as listeners with moderate cochlear hearing loss for speech understanding in steady background noise. These results were interpreted as suggesting that some good performing CI users and listeners with moderate hearing loss may access spectral information through ~8 independent frequency channels only. In others words, speech intelligibility in noise may be mostly limited by reduced frequency resolution in both subject groups. Therefore, it was expected that comparing CI and HI subjects on the STM detection test may be useful because both groups of listeners are believed to show reduced (and potentially comparable) frequency resolution. More specifically, we predicted that the effect of altered encoding of STM information would be greater for the higher spectral densities because of the reduced spectral resolution may have a greater impact on the processing of STM stimuli with higher spectral densities rather than lower spectral densities. Testing the three different subject groups also provided an opportunity to examine a possible relationship between STM detection performance and speech perception and other psychoacoustic performance both across the NH, HI, and CI subject groups.

## Methods

### Subjects

For the main test battery, three different subject groups participated in this study, including NH subjects, HI subjects, and CI subjects. Subjects who participated in the main test battery were native Korean speaking adults. The main test battery was performed in a double-walled sound-attenuating booth (IAC) at the Hearing Research Laboratory located in the Samsung Medical Center (Seoul, Korea). For these subjects, the use of human subjects and the experimental protocols were reviewed and approved by the Samsung Medical Center Institutional Review Board (2013-06-031). Every participant provided their written informed consent to participate in this study, and the Samsung Medical Center Institutional Review Board approved this consent procedure. Ten NH subjects (7 females and 3 males), ranging in age from 23 to 34 years (mean = 27.8 years), showed audiometric detection thresholds less than 20 dB HL at all audiometric frequencies between 250 and 8000 Hz in both ears. Twenty two HI subjects (10 females, 12 males) ranging in age from 21 to 71 years participated (mean = 46.6 years). HI subjects had a varying degree of hearing loss, ranged from moderate to moderate-to-severe. Pure-tone thresholds for tested ears for each HI subject are shown in Table 1. Mean pure-tone average (PTA) for 0.5, 1, 2, and 3 kHz averaged across 22 HI subjects was 53 dB HL.

**Table 1:** Audiometric thresholds for hearing-impaired (HI) subjects in dB HL.

Subject	Age (yrs)	Test Ear	250 Hz	500 Hz	1000 Hz	2000 Hz	3000 Hz	4000 Hz	8000 Hz
HI2	39	R	55	50	50	50	60	55	65
HI3	26	R	55	50	55	50	45	60	60
HI4	66	L	30	30	30	25	20	35	55
HI5	56	L	40	45	50	60	50	50	70
HI6	45	L	50	50	50	60	60	60	60
HI7	60	R	30	30	35	30	30	30	60
HI8	59	L	50	50	55	50	35	40	30
HI9	54	L	60	60	65	60	50	55	65
HI10	26	L	55	60	60	60	60	55	65
HI11	62	R	75	65	55	55	60	70	80
HI12	47	R	30	45	50	60	60	55	55
HI13	21	R	40	60	60	60	60	60	100
HI14	72	L	65	60	55	50	50	50	70
HI15	65	R	70	65	50	50	45	40	50
HI16	60	R	60	50	55	60	70	75	90
HI17	60	L	50	55	65	70	70	70	75
HI18	23	R	45	40	65	70	55	60	55
HI19	21	L	0	10	55	90	90	100	100
HI20	25	R	50	50	60	65	65	60	70
HI21	51	L	25	40	65	70	70	80	105
HI22	30	R	35	35	45	50	50	55	55
HI23	49	R	70	60	55	50	55	60	65

For the CI group, 23 unilateral CI patients (15 females, 8 males) ranging in age from 24 to 72 years participated (mean = 51.2 years). Thirteen CI subjects were implanted with the HiRes90K HiFocus implants manufactured by the Advanced Bionics. Seven CI subjects were users of the implant devices manufactured by the Cochlear Ltd. (4 subjects with Freedom implants; 2 subjects with CI422 implants; 1 subject with CI24RE implant). The remaining three subjects were implanted with the Flex implants manufactured by the MED-EL. Individual CI subject’s demographic information is provided in Table 2.

**Table 2:** Cochlear implant (CI) subject demographics. Subjects labeled with asterisks (CI24-CI29) participated in the experiment where test-retest reliability for the STM detection test was examined.

Subject ID	Age (yrs)	Duration of CI use (months)	Sound processor	Implant type	Sound coding strategy
CI01	43	75	Harmony	HiRes 90K HiFocus	Fidelity 120
CI02	24	59	Harmony	HiRes 90K HiFocus	HiResolution
CI03	66	94	Auria	HiRes 90K HiFocus	HiResolution
CI04	50	93	Auria	HiRes 90K HiFocus	HiResolution
CI05	54	98	Auria	HiRes 90K HiFocus	HiResolution
CI06	59	86	Freedom	CI24RE (CA)	ACE
CI07	72	74	Harmony	HiRes 90K HiFocus	Fidelity 120
CI08	49	53	Freedom	Freedom (Contour Advance)	ACE
CI09	68	33	OPUS2	Flex (soft)	FS4
CI10	55	95	Auria	HiRes 90K HiFocus	HiResolution
CI11	61	29	Nucleus 5	Freedom (Contour Advance)	ACE
CI12	56	32	N5	CI422	ACE
CI13	55	54	Harmony	HiRes 90K HiFocus	HiResolution
CI14	27	82	Harmony	HiRes 90K HiFocus	HiResolution
CI15	51	17	Nucleus 5	Freedom (Contour Advance)	ACE
CI16	58	24	OPUS2	Flex (soft)	FS4
CI17	65	11	Harmony	HiRes 90K HiFocus	Fidelity 120
CI18	24	86	Auria	HiRes 90K HiFocus	HiResolution
CI19	68	55	Freedom	Freedom (Contour Advance)	ACE
CI20	58	20	CP810	CI422	ACE
CI21	49	83	Auria	HiRes 90K HiFocus	HiResolution
CI22	24	28	OPUS2	Flex (soft)	FSP
CI23	42	95	Auria	HiRes 90K HiFocus	HiResolution
*CI24	56	96	Nucleus 5	Freedom	ACE
*CI25	86	4	Nucleus 5	Freedom	ACE
*CI26	72	8	Nucleus 5	Freedom	ACE
*CI27	57	7	Nucleus 5	Freedom	ACE
*CI28	43	8	Nucleus 5	Freedom	SPEAK
*CI29	57	9	Nucleus 5	Freedom	ACE

A separate group of 6 CI subjects (CI24-CI29) were tested at the University of Tennessee to evaluate learning effects and test-retest reliability for the STM detection test. All testing procedures for these additional 6 CI subjects followed the regulations approved by the University of Tennessee Health Science Center’s Institutional Review Board. These 6 CI subjects repeated the test and did two sets of six runs on separate days. Thresholds were determined by averaging the threshold from six runs.

* subjects tested at the University of Tennessee

### Test battery administration

A MATLAB (The Mathworks, Natick) graphical user interface running on a PC was used to present acoustic stimuli to subjects. For NH subjects, stimuli were presented monaurally through an ear insert phone at an average level of 65 dBA. For HI subjects, a frequency independent gain equal to half the PTA was applied to stimuli. With this gain, stimuli were generally presented at a most-comfortable level for HI subjects. The amplified stimuli were then presented monaurally through an insert ear phone. For CI subjects, stimuli were presented through a loud speaker (HS-50M, Yamaha, Japan) in the sound-field at an average level of 65 dBA. CI subjects sat at 1-m from the loudspeaker, and were asked to face it during the course of the experiment. All three groups of subjects participated in all psychoacoustic and speech perception tests. In addition to the STM detection test, a test of spectral modulation detection [9, 11, 12, 33] and temporal modulation detection [26]; a test of speech recognition in quiet [31]; and an assessment of sentence recognition in noise [32] was conducted. The order of test administration was varied within and across subjects.

### Spectrotemporal modulation (STM) detection test

To create STM stimuli with a bandwidth of four octaves (i.e. 354–5664 Hz), the following equation was used based on the previously established technique [27]:

S(x,t)=A×sin[2π×(ωt+Ωx)+Φ], (1)

in which *x* is the position on the logarithmic frequency axis in octaves (i.e. *x* = *log*_2_(*f*/354), here *f* is frequency), and *t* is time on the time axis. Four thousands carrier tones were spaced equally on a logarithmic frequency scale with a bandwidth of 354–5656 Hz. The stimuli had 1 sec total duration. The spectral envelope of the complex tones was modulated as a single sinusoid along the logarithmic frequency axis on a linear amplitude scale. In Eq (1), *A* is the amplitude of the rippled spectral modulation amplitudes, which is defined relative to the flat spectrum. When *A* was set to a value between 0 and 1, it corresponded to 0 to 100% spectral modulation of the flat ripple envelope. Ω is the spectral density in units of cycles per octave (c/o). Φ is the spectral modulation starting phase in radians for carrier tones that were randomized in radians (ranged between 0 to 2π). The STM stimuli were also modulated in time by having the modulated spectral envelopes sweep across the frequency at a constant velocity. In Eq (1), *ω* sets spectral modulation velocity as the number of the sweeps per second (Hz), which is referred to as temporal rate in the current study. The positive and negative velocity constructs the STM stimuli with spectral modulations (or frequency modulations) that either rise or fall in frequency and repeat over time. As Bernstein et al. (2013) [29] showed no effect of the direction of spectral modulation on STM detection thresholds for NH and HI listeners, the current study tested a falling direction of spectral modulation alone.

To measure STM detection thresholds, a 2-interval, 2-alternative adaptive forced-choice (2I, 2-AFC) paradigm was used. A silence interval of 500 ms was used between the two intervals. One of the intervals consisted of modulated noise (i.e. test signal), and the other interval consisted of steady noise (i.e. reference signal). Subjects were instructed to choose an interval containing sound like bird-chirping, vibrating, or moving over time and frequency. Subject’s task was to identify the interval which contained a STM stimulus. A 2-down, 1-up adaptive procedure was used to measure STM detection thresholds, starting with a modulation depth of 0 dB and decreasing in steps of 4 dB from the first to the fourth reversal, and 2 dB for the next 10 reversals. For each testing run, the final 10 reversals were averaged to obtain the STM detection threshold. In order to evaluate STM detection performance for different modulation conditions, three different spectral densities (Ω = 0.5, 1, and 2 c/o) and two different temporal rates (*ω* = 5 and 10 Hz) were tested. Thus, a total of six different sets of STM stimuli were tested. Subjects completed all these six different stimulus conditions in random order, and then subjects repeated a new set of six stimulus conditions with a newly created random order. The sequence of stimulus conditions was randomized within and across subjects. A third adaptive track was obtained if the difference between the first two tracks exceeded 3 dB for a given stimulus condition. The final threshold for each STM stimulus condition was the mean of these two (or three) adaptive tracks. Before actual testing, experimenters played example stimuli for subjects until they became familiar with the STM stimuli and task. During this practice run, subjects were able to hear and compare two different stimuli (modulated vs. unmodulated) as many as they want until they fully understand the task.

### Spectral modulation detection test

Spectral modulation detection performance was evaluated using a spectral-ripple detection paradigm [9, 11, 12, 33]. To create static-ripple stimuli, 2555 tones were spaced equally on a logarithmic frequency scale with a bandwidth of 354–5656 Hz. The ripple peaks and valley were spaced equally on a logarithmic frequency scale with a ripple density of 1 c/o. A ripple density of 1 c/o was tested to examine a potential relationship with detection thresholds for STM stimuli with a spectral density of 1 c/o. The spectral modulation starting phase for ripple stimuli was randomly selected from a uniform distribution (0 to 2π rad). The stimuli had 500 ms total duration. Spectral modulation detection thresholds were determined using a three-interval, three-alternative forced choice (3-I, 3-AFC), similar to the method reported by Anderson et al. (2012). As discussed in Introduction, it should be noted that static-ripple detection in the current study is different from spectral-ripple discrimination that has been widely used in the literature [3–5, 7, 8, 12, 36, 37]. In the current study, for each set of three intervals, two intervals contained the unmodulated broadband noise, and the test interval, chosen at random with equal *a priori* probability on each trial, contained the static-ripple stimulus. An inter-stimulus-interval of 500 msec was used between intervals. Stimuli were equated to the same root-mean-square level and a level rove of ±2 dB (in 1-dB increments) was randomly selected for each interval in the three-interval task. Three numerically labeled virtual buttons were displayed on the computer screen, corresponding to the three intervals, and subjects were instructed to click on the button corresponding to the interval (i.e. static-ripple stimulus) that sounded different from two others. Visual feedback was provided after each trial to indicate the interval that presented the static-ripple stimulus. For each trial, fresh unmodulated and rippled noise stimuli were used. Each test run began with a peak-to-valley ratio for the rippled stimulus of 20 dB, with which most subjects were easily able to detect the spectral modulation. The spectral modulation depth was varied adaptively in a two-down, one-up adaptive procedure. After each incorrect response, the spectral modulation depth was increased by a step, and it was decreased after two correct consecutive responses. This procedure tracks the peak-to-valley ratio that could be detected with an accuracy of 70.7% correct [38]. The initial step size was 2 dB for the first four reversals. The step size was then changed to 0.5 dB for the remaining ten reversals. Spectral modulation detection threshold was defined for each run as the arithmetic mean of the peak-to-valley ratios at the final ten reversal points.

### Temporal modulation detection test

The temporal modulation detection test was administered as previously described by Won et al. (2011) [26]. For the modulated stimuli, sinusoidal amplitude modulation was applied to the wideband noise carrier. The stimulus duration for both modulated and unmodulated signals were 1 second. Modulated and unmodulated signals were gated on and off with 10 ms linear ramps, and they were concatenated with no gap between the two signals. The temporal modulation detection threshold was measured using a 2-interval, 2-alternative adaptive forced-choice (2I, 2-AFC) paradigm. One of the intervals consisted of modulated noise, and the other interval consisted of steady noise. Subject’s task was to identify the interval which contained the modulated noise. A modulation frequency of 10 Hz was tested to examine a potential relationship with detection thresholds for STM stimuli with 10 Hz temporal rate. A 2-down, 1-up adaptive procedure was used to measure the modulation depth threshold, starting with a modulation depth of 100% and decreasing in steps of 4 dB from the first to the fourth reversal, and 2 dB for the next 10 reversals. For each testing run, the final 10 reversals were averaged to obtain the modulation detection threshold (MDT). MDTs in dB relative to 100% modulation (i.e. 20log_10_(*m*_*i*_)) were obtained, where *m*_*i*_ indicates the modulation index. The threshold for each subject was calculated as the mean of three testing runs.

### Sentence recognition in quiet

Two lists of Korean Central Institute for the Deaf (K-CID) sentences [31] were administered. Each list contained ten sentences with four key words, for a total of 80 key words were scored for each subject. All participants were instructed to verbally repeat the sentence they heard. A total percent correct score was calculated as the percent of key words correctly recognized.

### Sentence recognition in noise

A Korean version of HINT (Hearing in Noise Test) sentences [32] was administered in the presence of background noise. The level of steady-state background noise was fixed at 55 dBA. The level of target sentences was varied using a 1-up, 1-down adaptive procedure to estimate speech reception threshold (SRT) at 50% correct performance. Subjects completed two test runs. An average SRT across the two test runs are reported.

### Data analysis

For the STM detection test, mean detection thresholds for each stimulus condition are reported. We conducted a mixed between-within subjects repeated measures analysis of variance (ANOVA) to compare performance with hearing mechanism (NH, HI, and CI) and subjects’ ages as the between-subject factors and the STM stimulus conditions as the within-subject factors. Here, the mixed between-within subjects repeated measures ANOVA was used to address the main question to determine if altered encoding of STM cues due to the hearing-loss (HI group) or due to the CI-auditory nerve interface (CI group) affects STM detection performance; if so, a post-hoc paired samples t-test was further performed to systematically compare performance for each modulation condition among the three subject groups. Subject ages were also included as the between-subject factor because in the current study, NH group had a smaller range of ages (i.e., 11 years) compared to HI and CI groups (50 and 48 years, respectively).

As mentioned in Introduction, we hypothesized that the effect of altered encoding of STM information would be greater for the higher spectral densities because of the reduced spectral resolution associated with hearing loss or CI processing. It was also predicted that the effect of altered encoding of STM information would be similar between 5 and 10 Hz temporal modulation rates, because at these relatively lower temporal rates, the altered encoding of temporal modulation information has previously shown little effect on temporal modulation detection performance for HI and CI listeners [22, 26, 39].

Correlations of the STM detection test with the other tests were assessed using a Pearson’s linear correlation coefficient. Furthermore, a partial correlation analysis controlling for the effect of either the static-ripple detection test or temporal modulation detection test was performed to determine if the STM detection test is still predictive of speech perception before and after the control.

## Results

### STM detection performance for NH, HI, and CI subjects

**Fig 3:**
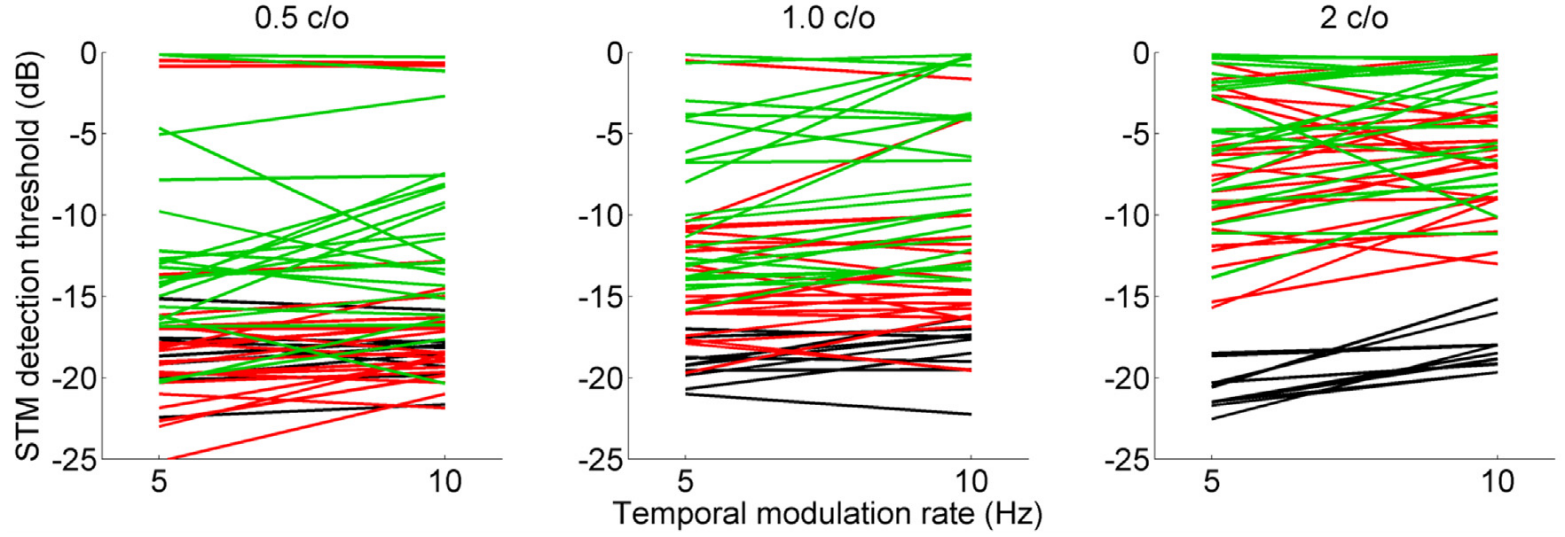
STM detection thresholds for individual subjects, shown as a function of temporal rate. Results for spectral rates of 0.5, 1.0, and 2.0 c/o are shown in the left, middle, and right column, respectively. The black, red, and green lines represent the STM detection thresholds for normal-hearing, hearing-impaired, and cochlear implant subjects, respectively.

**Fig 4:**
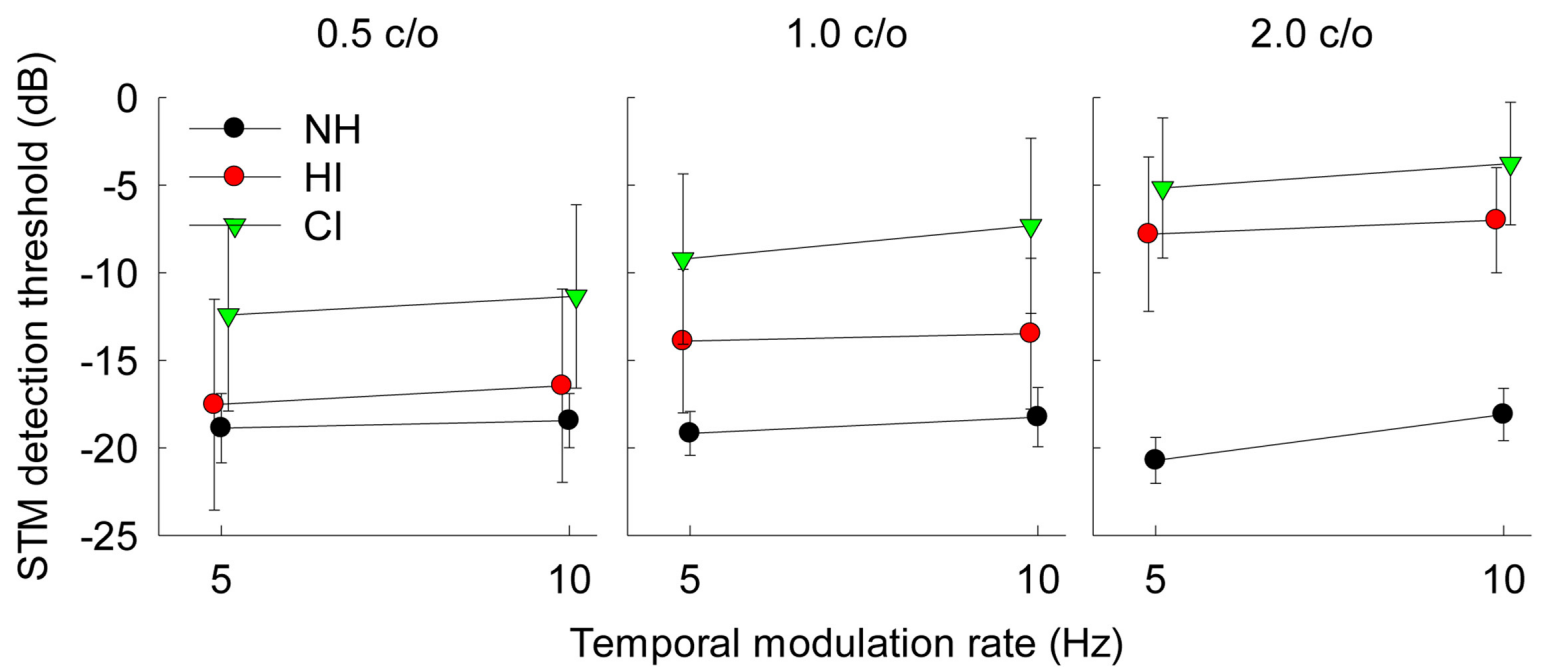
Mean STM detection thresholds averaged across subjects. Error bars represent one standard deviation across subjects.

Fig 3 shows STM detection thresholds for individual subjects as a function of temporal rate. Results for three different spectral densities, 0.5, 1.0, and 2.0 c/o are shown in the left, middle, and right column, respectively. The black, red, and green lines represent NH, HI, and CI subjects, respectively. More negative STM thresholds indicate better detection performance. Fig 4 shows mean STM detection thresholds averaged across 10 NH subjects, 22 HI subjects, and 23 CI subjects for six different stimulus conditions. Error bars represent one standard deviation across subjects. Overall, NH subjects showed the best STM detection performance with a range between -18.1 and -20.7 dB; while CI subjects showed the poorest STM detection performance with a range between -3.8 and -12.4 dB. HI subjects showed performance between those of the NH and CI subjects with a range between -6.7 and -17.4 dB. For HI and CI subject groups, STM detection thresholds generally increased (i.e. performance decreased) as a spectral density increased from 0.5 to 2.0 c/o. While the effect size was generally small, there was a trend that STM detection thresholds increased as a temporal rate increased from 5 to 10 Hz. Of particular interest, a wide range of detection performance was observed at each stimulus condition across individual subjects (Fig 3). At a spectral density of 0.5 c/o, STM detection thresholds for CI subjects strongly overlapped with thresholds for HI subjects. More interestingly, some CI subjects showed STM detection performance which overlapped with performance for NH subjects. At spectral densities of 1.0 and 2.0 c/o, STM detection thresholds for CI subjects still overlapped with STM thresholds for HI subjects.

A mixed between-within subjects ANOVA confirmed this pattern of results. The main effects of spectral density [F(2,26) = 90.0, *p* < 0.001] and temporal rate [F(1,13) = 14.2, *p* = 0.002] on STM detection thresholds reached significance. A two-way interaction between the effects of spectral density and temporal rate did not reach significance [F(2,26) = 1.94, *p* = 0.16]. Also, a two-way interaction between the subject group and temporal rate did not reach significance [F(2, 13) = 2.26, *p* = 0.14]. However, a two-way interaction between the subject group and spectral density reached significance [F(4,26) = 11.3, p < 0.001], indicating the potential influence of different frequency selectivity across the three subject groups on STM detection thresholds. Here, the significant two-way interaction between the subject group and spectral density supports our hypothesis that reduced frequency selectivity due to the hearing loss or the CI-auditory nerve interface would alter the encoding of STM information in a different manner for NH, HI and CI subjects.

To better understand which spectral density conditions gave rise to the significant two-way interaction between the subject group and spectral density, a post hoc independent samples t-test was performed on the STM data collapsed across temporal rates. This analysis was carried out to systematically compare performance for the NH, HI, and CI subjects for three different spectral densities to identify the specific spectral density where performance for the three subject groups differed significantly each other. Table 3 summarizes the results for these analyses. Overall, STM detection thresholds for NH subjects were significantly lower (i.e. better) than CI subjects. Comparing NH and HI subjects, only the spectral density of 2.0 c/o showed a significant difference in performance after a Bonferroni correction, which is consistent with Bernstein et al. (2013) [29], where HI subjects showed poorer STM detection performance compared to NH subjects for higher spectral densities but not lower spectral densities. Between HI and CI subjects, significant differences were shown in performance for 0.5 and 1.0 c/o, but not for a higher spectral density of 2.0 c/o.

**Table 3:** Results for post hoc independent samples t-tests on the STM detection thresholds collapsed across two temporal modulation rates. For each comparison, *p*-values are reported. Bold values indicate a significant difference after applying a Bonferroni correction.

Comparison	0.5 c/o	1.0 c/o	2.0 c/o
NH vs. HI	0.38	0.033	**0.004**
NH vs. CI	**< 0.001**	**< 0.001**	**< 0.001**
HI vs. CI	**0.003**	**< 0.001**	0.006

A three-way interaction between the subject group, spectral density, and temporal rate was examined. This analysis showed that the three-way interaction did not reach significance [F(4,26) = 0.99, *p* = 0.43]. Note that the two-way interaction between the subject group and temporal rate was not significant, but the two-way interaction between the subject group and spectral density was significant. Therefore, the non-significant three-way interaction, coupled with the significant two-way interaction between the subject group and spectral density, suggests that different frequency selectivity across the three subject groups may be the primary factor constraining STM detection performance differently for NH, HI and CI subjects.

It should be emphasized, however, that the temporal rates tested in the current study were very low. Therefore, a significant two-way interaction between the subject group and temporal rate would be expected if higher temporal rates were tested, as shown by Bernstein et al. (2013). Subsequently, it is possible to observe a significant three-way interaction between the subject group, spectral density, and temporal rate if a wider range of spectral density or temporal rate was administered for the STM detection test.

Finally, a potential influence of different age ranges for each subject group upon STM detection performance was examined. All possible two-way interactions (i.e., spectral density × age and temporal rate × age), three-way interactions (i.e., spectral density × subject group × age, temporal rate × subject group × age, and spectral density × temporal rate × age), and four-way interaction (i.e., spectral density × temporal rate × subject group × age) were considered. These analyses showed that none of these interactions reached significance, suggesting that it is unlikely that a different range of ages in each subject group affected STM detection performance.

### Learning effects and test-retest reliability

**Fig 5:**
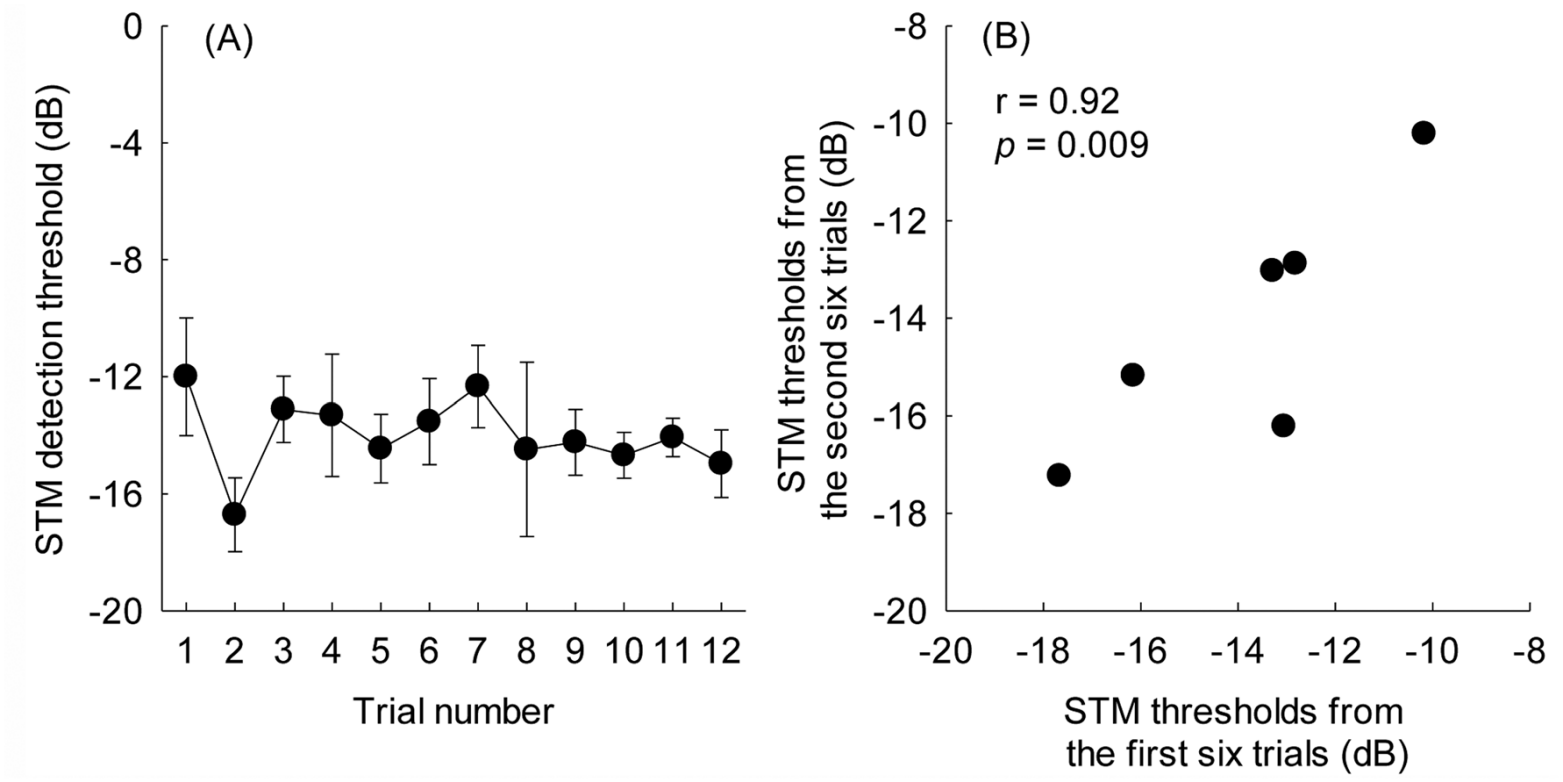
(A) Effects of learning for the STM detection test. The figure shows mean STM detection thresholds as a function of trial number for the 10 Hz and 0.5 c/o condition. Error bars show one standard error based on data from 6 CI subjects. (B) Reliability of the STM detection test. The relationship between the STM detection thresholds determined by the first six repetitions and the second six repetitions for 6 CI subjects are shown.

Learning effects and test-retest reliability were examined to determine if the STM detection test could serve as a reliable measure for CI outcomes. In order to reduce the testing and prevent any fatigue, a single STM stimulus condition (10 Hz and 0.5 c/o) rather than all six conditions was tested. Fig 5A shows the mean threshold for STM detection as a function of trial number, computing the mean at each repetition averaged across 6 CI subjects. There was a limited learning effect across 12 trials. In fact, there was not a statistically significant difference between STM detection thresholds for the 1^st^ and 12^th^ trial [t(5) = 1.99, *p* = 0.10]. A repeated-measures ANOVA also demonstrated that there was no effect of trial number [F(11, 55) = 1.06, *p* = 0.41] throughout the 12 STM detection tests. Fig 5B shows the average thresholds of the first six trials plotted against the average thresholds of the second six trials for 6 CI subjects. The average of the first six was -13.9 dB, and the average of the second six was -14.1 dB. A paired t-test revealed that there was no significant improvement between the first and second six trials (*p* = 0.67). The intraclass correlation between the thresholds from the first and second six trials was 0.92 (*p* = 0.009), which reveals promising test-retest reliability of the STM detection test.

### Performance for spectral and temporal modulation detection

**Fig 6:**
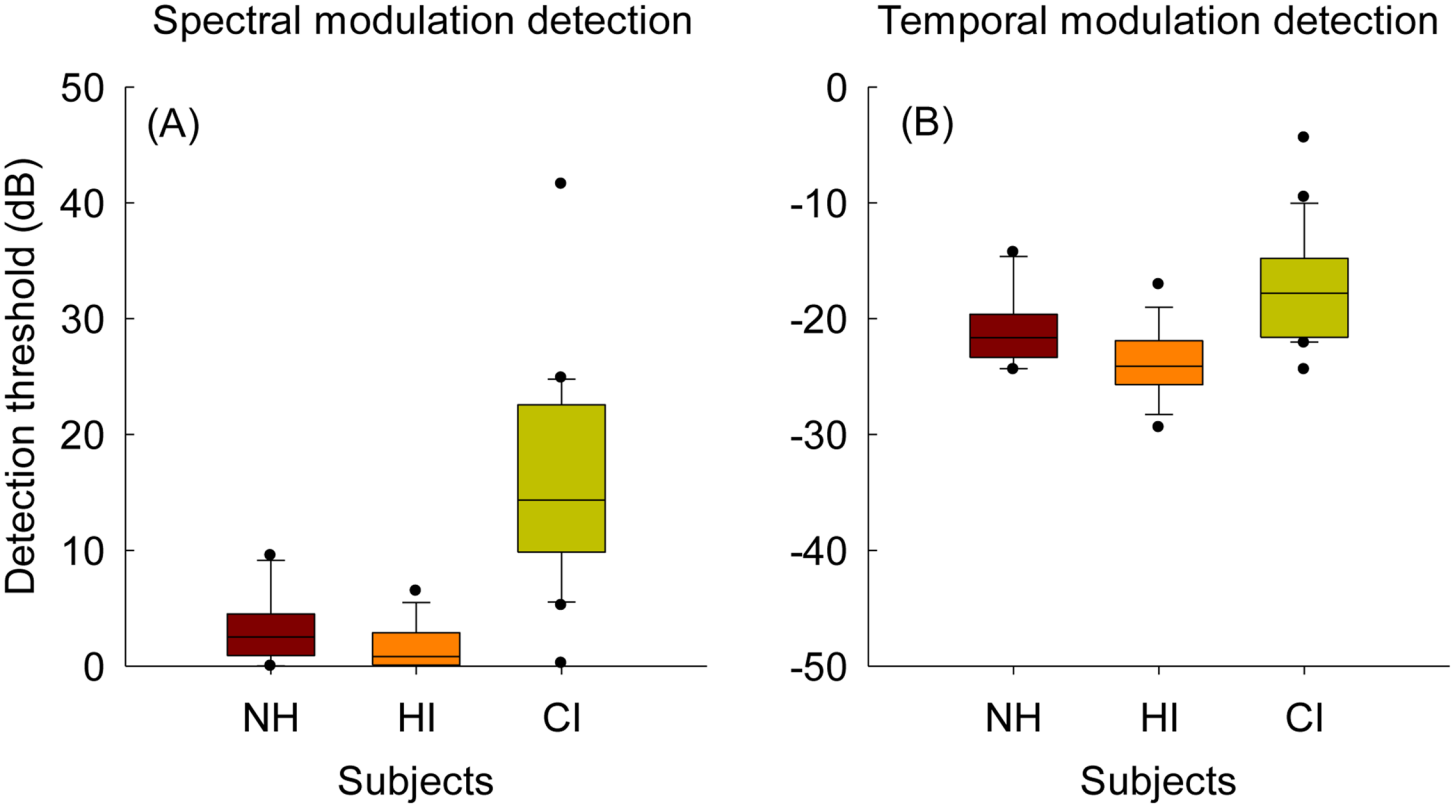
Boxplots for spectral modulation detection at 1 c/o (A) and temporal modulation detection at 10 Hz (B) for NH, HI, and CI subjects.

In Fig 6A, box-and-whisker plots for spectral modulation detection for the three subject groups are shown. Here, lower detection thresholds indicate better spectral modulation detection performance. NH and HI subjects showed similar performance on spectral modulation detection tested at a spectral density of 1 c/o [t(30) = 1.9, *p* = 0.065]. However, CI subjects performed significantly worse than both NH [t(29.7) = -6.1, *p* < 0.001] and HI subjects [t(24) = -7.6, *p* < 0.001] on the spectral modulation detection test.

Fig 6B shows box-and-whisker plots for the temporal modulation detection test at 10 Hz for the three different subject groups. In this plot, more negative detection thresholds imply better temporal modulation detection performance. After applying a Bonferroni correction, independent sample t-tests showed that there was no significant difference in temporal modulation detection performance between NH and HI subjects [t(30) = 2.7, *p* = 0.011] and between NH and CI subjects [t(31) = -2.2, *p* = 0.032]. However, HI subjects performed significantly better than CI subjects [t(35.4) = -5.8, *p* < 0.001].

### Correlations with other psychoacoustic and speech perception tests

**Fig 7:**
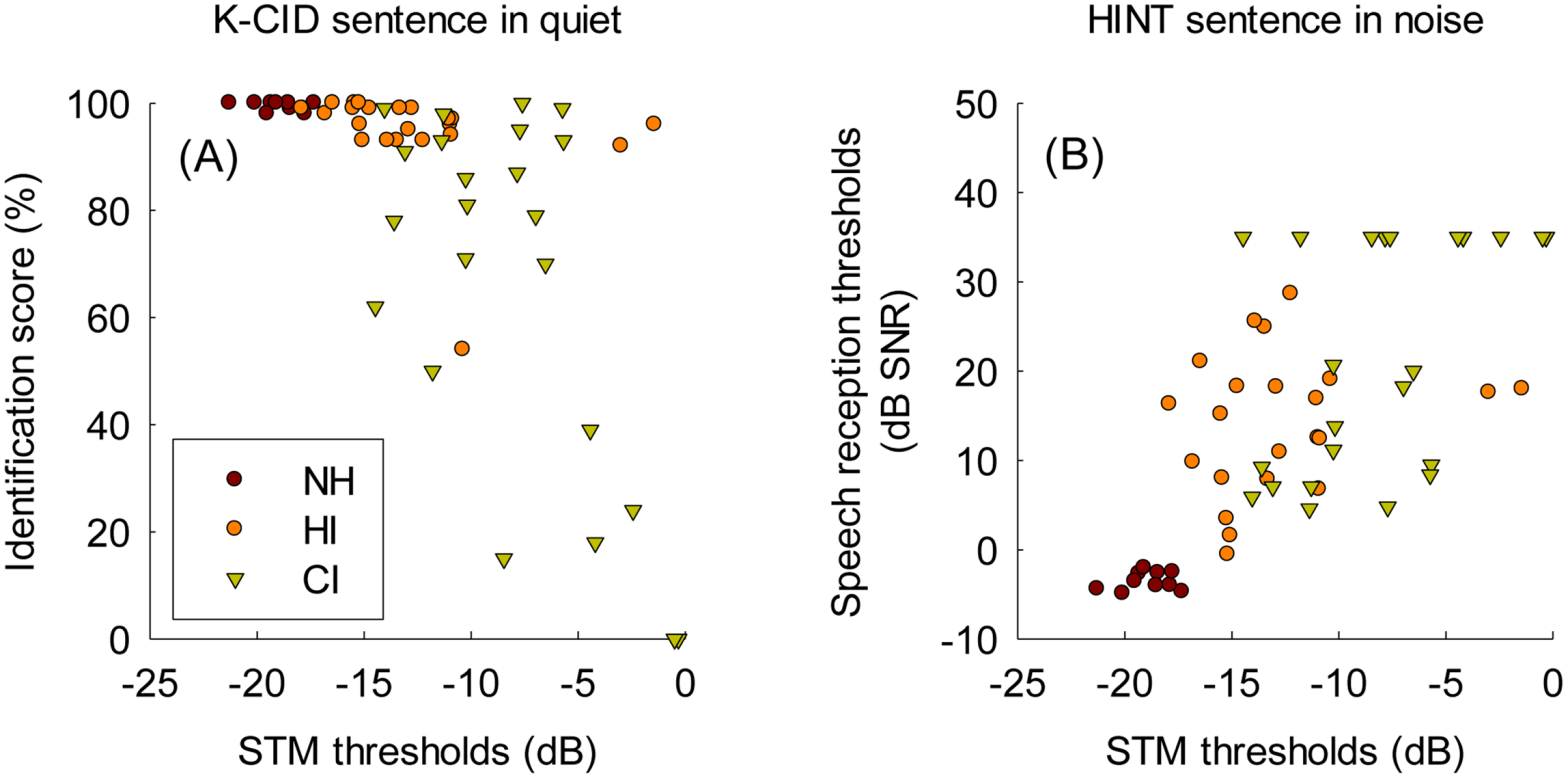
The relationship between mean STM detection thresholds averaged across the six different stimulus conditions and K-CID sentence recognition in quiet (A) and HINT sentence recognition in noise (B) across NH, HI, and CI subjects.

Fig 7 shows the relationship between STM detection performance and both sentence identification in quiet (i.e. K-CID test) and in noise (i.e. K-HINT test) across the three subject groups. For these analyses, mean STM thresholds averaged across the six different stimulus conditions were used. A significant relationship was found between mean STM detection thresholds and both sentence recognition in quiet (r = -0.63, *p* < 0.001) and in noise (r = 0.67, *p* < 0.001). These results suggest that, across all three different subject groups, the encoding of STM cues may be an important factor to contribute to speech perception abilities. Note that when controlling the effects of subjects’ ages on these correlation analyses, the partial correlations still stayed significant, suggesting that subjects’ ages did not affect the relationship between STM detection performance and sentence recognition in quiet and noise.

**Fig 8:**
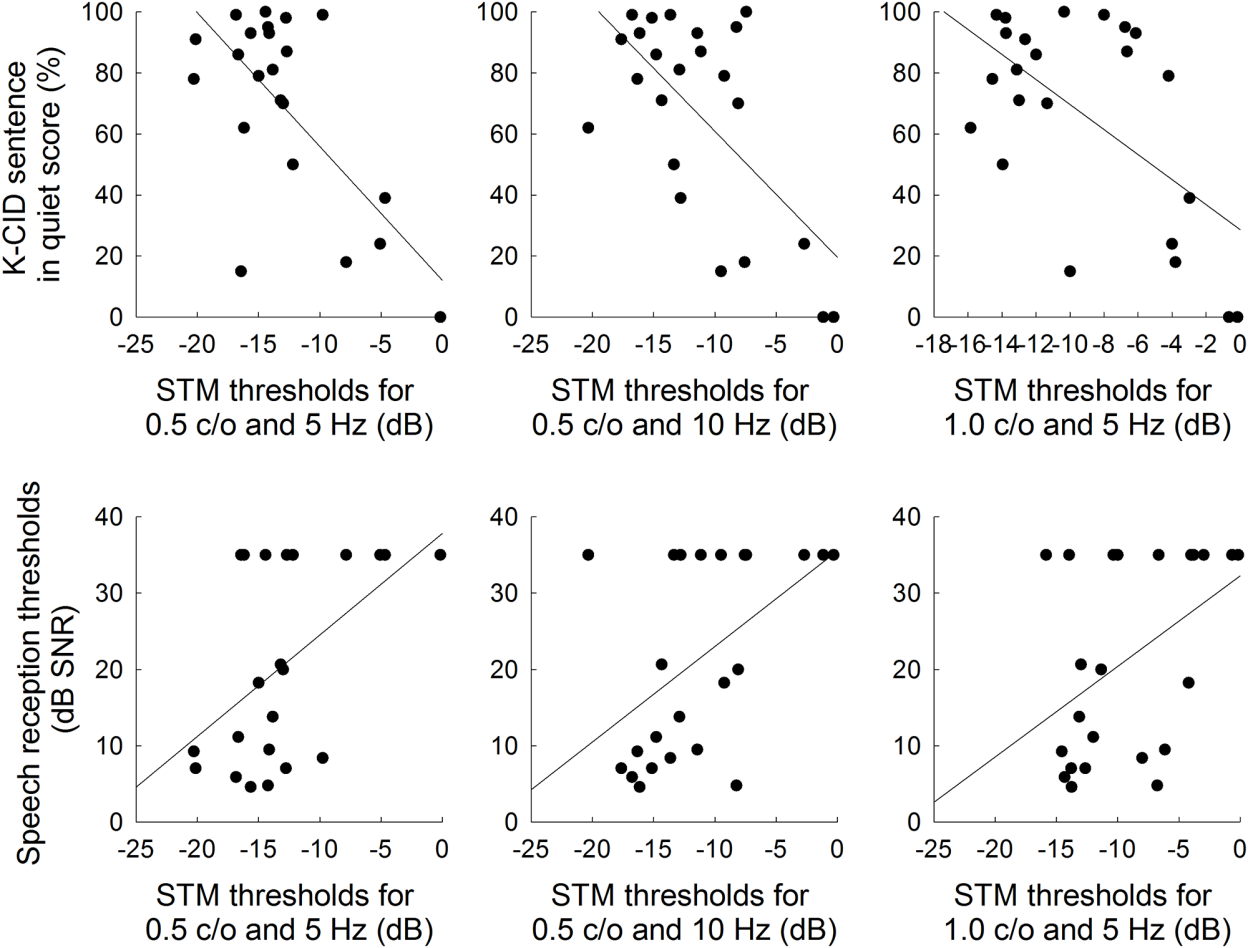
Relationship between STM detection and K-CID sentence recognition in quiet (upper panel) and HINT sentence recognition in noise (lower panel) for CI subjects. Linear regressions are represented by the solid lines.

The relationship among the STM detection performance, speech perception abilities, and other psychoacoustic sensitivities were further examined for CI subjects. For these analyses, each of all six STM stimulus conditions along with mean STM thresholds were used to evaluate correlations with sentence recognition in quiet and noise, as summarized in Table 4. Significant correlations at the level of 0.05 are shown in bold. Fig 8 shows scatter-plots for the relationship between STM detection thresholds and K-CID sentence recognition in quiet (upper panel) and HINT sentence recognition in noise (lower panel) in CI subjects. The results showed that the K-CID sentence recognition scores in quiet were significantly correlated with STM detection thresholds for lower spectral densities of 0.5 and 1.0 c/o regardless of temporal modulation rates. These STM detection thresholds for 0.5 and 1.0 c/o (except for 1.0 c/o & 10 Hz) were also significantly predictive of the HINT sentence recognition performance in noise. Mean STM thresholds significantly correlated with both K-CID and HINT scores. Note that in the current study, however, the Bonferroni corrections were not applied due to the increased risk of a type II error for the number of comparisons made (e.g., [40], as cited in [37, 41]). Instead, we provide all correlation coefficients and their associated *p*-values.

**Table 4:** Correlations of STM detection test measures with sentence recognition in quiet (K-CID) and in noise (K-HINT), spectral modulation detection (SMD) and temporal modulation detection (TMD) tests in CI subjects. R is the Pearson correlation coefficient, and *p* is the significance. Bold values indicate significant correlations at the level of 0.05. The asterisks indicate the conditions where the correlations became no longer significant after factoring out the effects of subjects’ ages.

	0.5 c/o & 5 Hz		0.5 c/o & 10 Hz		1.0 c/o & 5 Hz		1.0 c/o & 10 Hz		2.0 c/o & 5 Hz		2.0 c/o & 10 Hz		Mean STM	
	R	*p*	R	*P*	R	*P*	R	*p*	R	*p*	R	*P*	R	*p*
K-CID	**-0.71**	**<0.001**	**-0.64**	**0.001**	**-0.59**	**0.003**	-0.43*	**0.039**	-0.41	0.054	-0.41	0.052	**-0.62**	**0.002**
K-HINT	**0.56**	**0.005**	**0.51**	**0.014**	0.44*	**0.034**	0.36	0.096	0.41	0.055	0.12	0.59	**0.47**	**0.022**
SMD at 1 c/o	0.30	0.16	0.39	0.063	**0.47**	**0.024**	0.33	0.12	0.39	0.066	0.40	0.059	**0.43**	**0.041**
TMD at 10 Hz	0.30	0.17	0.21	0.35	0.18	0.41	0.34	0.16	0.14	0.52	0.39	0.064	0.29	0.19

Relationships between spectral modulation detection at 1.0 c/o and STM detection thresholds for each STM stimulus condition were also examined. For these analyses, we predicted that spectral modulation detection at 1.0 c/o would be correlated with STM detection thresholds for the same spectral density. This prediction was supported by the significant correlation found between spectral modulation detection thresholds at 1.0 c/o and STM detection thresholds at 1.0 c/o and 5 Hz (r = 0.47, *p* = 0.024). Note that spectral modulation detection thresholds at 1.0 c/o were not correlated with the STM detection thresholds for the rest of five STM stimulus conditions. Similarly, correlations between temporal modulation detection thresholds at 10 Hz and STM detection thresholds were examined with a prediction that STM thresholds at 10 Hz would show significant relationship with temporal modulation detection at 10 Hz. In contrast with the results for spectral modulation detection, temporal modulation detection at 10 Hz was not predictive of STM detection performance for all six STM stimulus conditions.

Previous studies have shown that spectral modulation detection [11, 12, 33] and temporal modulation detection [16, 24, 26] are predictive of speech perception for CI users. To better understand the nature of the relationship between STM detection performance and speech perception abilities for CI subjects, we performed partial correlations controlling for the contribution of either spectral or temporal modulation detection. Tables 5 and 6 summarizes the results for these partial correlation analyses. In Table 5, when the effect of spectral modulation detection at 1 c/o was factored out, the correlations between (1) STM thresholds for 1.0 c/o & 10 Hz and K-CID scores, (2) STM thresholds for 1.0 c/o & 5 Hz and K-HINT scores, and (3) mean STM thresholds and K-HINT scores became no longer significant. In contrast, when the effect of temporal modulation detection at 10 Hz was controlled for (Table 6), the partial correlation analyses produced little change in correlation coefficients, and more importantly, did not change the significance at the 0.05 level. Taken together, these analyses suggest that detection abilities for slow spectral modulation patterns rather than temporal modulation patterns might have played a primary role for the relationship between STM detection performance and speech perception abilities for CI subjects.

**Table 5:** Results for partial correlations analyses. Bold values indicate significant correlations at the level of 0.05. Underlined italics indicate the correlations that were originally significant shown in Table 4 but became non-significant after controlling for predictive effect of static-ripple detection.

Partial correlations between STM detection test measures and sentence recognition in quiet (K-CID) and in noise (K-HINT) while controlling for predictive effect of static-ripple detection.															
	0.5 c/o & 5 Hz		0.5 c/o & 10 Hz		1.0 c/o & 5 Hz		1.0 c/o & 10 Hz		2.0 c/o & 5 Hz		2.0 c/o & 10 Hz		Mean STM	
	R	*p*	R	*P*	R	*p*	R	*p*	R	*p*	R	*p*	R	*p*
K-CID	**-0.68**	**0.001**	**-0.57**	**0.006**	**-0.48**	**0.024**	*-0*.*34*	*0*.*12*	-0.28	0.20	-0.28	0.20	**-0.53**	**0.011**
K-HINT	**0.51**	**0.015**	**0.43**	**0.048**	*0*.*34*	*0*.*13*	0.27	0.22	0.31	0.16	-0.024	0.92	*0*.*38*	*0*.*08*

**Table 6:** Results for partial correlations analyses. Underlined italics indicate the correlations that were originally significant shown in Table 4 but became non-significant after controlling for predictive effect of temporal modulation detection.

Partial correlations between STM detection test measures and sentence recognition in quiet (K-CID) and in noise (K-HINT) while controlling for predictive effect of temporal modulation detection.															
	0.5 c/o & 5 Hz		0.5 c/o & 10 Hz		1.0 c/o & 5 Hz		1.0 c/o & 10 Hz		2.0 c/o & 5 Hz		2.0 c/o & 10 Hz		Mean STM	
	R	*p*	R	*P*	R	*p*	R	*p*	R	*p*	R	*p*	R	*p*
K-CID	**-0.75**	**<0.001**	**-0.66**	**0.001**	**-0.60**	**0.003**	**-0.46**	**0.033**	-0.41	0.057	**-0.45**	**0.037**	**-0.65**	**0.001**
K-HINT	**0.62**	**0.002**	**0.54**	**0.01**	**0.47**	**0.027**	0.41	0.062	0.43	0.048	0.17	0.44	**0.53**	**0.012**

## Discussion

The current study was designed to evaluate CI users’ sensitivity to both spectral and temporal acoustic cues *together* using STM stimuli and investigate how STM detection performance relates to sentence identification performance in quiet and noise. This approach differs from previous studies where spectral modulation sensitivity [3–5, 7, 10–12] or temporal modulation sensitivity [16, 18, 23, 24, 26] was measured *separately* and the relationship with speech perception abilities in CI users was assessed. Despite the methodological difference, the findings from the current study are largely consistent with previous studies that spectral and temporal modulation sensitivities are important factors affecting speech perception outcomes for CI users.

### A. Altered encoding of STM cues through cochlear implants

Overall, the results for the STM detection test indicate that altered encoding of STM cues due to the CI-auditory nerve interface for the CI group and the effect of sensorineural hearing loss for the HI group degraded STM detection performance compared to the NH group. Within each group, a wide range of performance for STM detection was observed across the HI and CI subjects (Fig 3). Interestingly, variability in STM detection performance was also observed in NH subjects. Although the effect size was relatively small, all three subject groups showed a change in STM detection thresholds when the temporal modulation rate increased from 5 to 10 Hz. The fact that the two-way interaction between the effects of temporal rate and the subject group did not reach significance [F(2.52) = 1.1, *p* = 0.34], suggesting that NH, HI and CI subjects showed a similar increase in STM detection thresholds as the temporal modulation rate increased from 5 to 10 Hz. This result contrasts with Bernstein et al. (2013) [29], where a significant interaction was found between the effects of temporal rates and hearing loss on STM detection thresholds. A potential reason for this difference may be that we used relatively slow modulation rates up to 10 Hz, whereas Bernstein et al. [29] tested 4, 12, and 32 Hz of temporal modulation rates. At slow temporal modulation rates such as 5 and 10 Hz, HI or CI subjects typically show good performance relative to NH subjects [18, 22, 24, 26, 37]. If a higher temporal rate was tested such as 32 Hz or beyond, HI and CI subjects would have shown more degradation in STM detection compared to the performance at 10 Hz and produced a potential two-way interaction between temporal rate and subject group.

Spectral densities showed a significant effect [F(2,104) = 77.8, *p* < 0.001] on STM detection performance across the three subject groups. NH subjects showed relatively consistent performance across the three spectral densities. However, STM detection performance decreased markedly for HI and CI subjects as the spectral density increased from 0.5 to 2.0 c/o. Comparisons between the subject groups (Table 3) revealed that CI subjects showed significantly poorer STM detection performance for all three spectral densities than NH subjects. However, CI subjects showed significantly poorer detection performance relative to HI subjects only for 0.5 and 1.0 c/o but not for 2.0 c/o. In contrast, the difference in STM detection performance between NH and HI subjects was significant only for 2.0 c/o but not for 0.5 and 1.0 c/o, which is consistent with Bernstein et al. (2013) [29]. These results may imply that poor frequency selectivity in HI and CI subjects may constrain their STM detection performance, but the extent to which frequency selectivity constrains STM detection abilities may partly depend on the spectral densities for the test signals. Furthermore, a two-way interaction between the subject group and spectral density reached significance [F(4,104) = 21.8, *p* < 0.001]. In sum, the finding that the two-way interaction of the subject group was significant with spectral density, but not with temporal rate may suggest that different frequency selectivity across the three subject groups might have played a primary role to constrain the pattern of STM detection thresholds for the range of stimulus conditions tested in the current study in a different manner for NH, HI, and CI subjects.

### B. Spectral and temporal modulation detection performance for three subject groups

In the current study, spectral modulation and temporal modulation detection performance were also measured for the three different subject groups. There was no significant difference between NH and HI subjects with respect to spectral (Fig 6A) and temporal modulation detection (Fig 6B) performance. CI subject showed significantly poorer performance than NH subjects for the spectral modulation detection, but the difference in temporal modulation detection thresholds between these two groups was not significant after applying a Bonferroni correction. CI subjects showed significantly poorer performance on both spectral and temporal modulation detection than HI subjects.

These results, however, should be interpreted with caution because differences in performance on spectral and temporal modulation detection between subject groups strongly depend on signal configurations (e.g., modulation frequency). For example, Henry et al. (2005) [5] showed that CI users’ spectral resolution measured by spectral-ripple discrimination is worse than that of NH and HI subjects, which is consistent with the results for the spectral modulation detection in the current study. However, in Henry et al. [5], HI subjects showed poorer performance on spectral-ripple discrimination than NH subjects, whereas in the current study, NH and HI subjects showed comparable performance on the spectral modulation detection. It should be noted that, however, the degrees of hearing loss for HI subjects in the current study ranged primarily between moderate and moderate-to-severe. As noted in the method, the mean PTA averaged across 23 HI subjects was 53 dB HL. A recent study [42] by the University of Iowa demonstrated that there is no significant difference in spectral ripple discrimination performance for recently implanted subjects (implanted during the last 10 years) compared to HI listeners. Therefore, the significant difference in spectral modulation detection performance between HI and CI subjects may be partly due to the degree of hearing loss for HI subjects.

In this study, a relatively slow temporal modulation frequency was used for the temporal modulation detection test. Previously, listeners with sensorineural hearing loss have shown comparable performance on temporal modulation detection compared to NH subjects [22, 37, 43] as a result of the loudness recruitment that gives rise to the loss of the fast-acting mechanism in the cochlea and the enhancement of amplitude modulation cues in the auditory periphery.

At 10 Hz modulation frequency, NH and HI subjects in the current study also showed similar performance on the temporal modulation detection test. In fact, when stimuli were presented at a most comfortable level for HI subjects, there was a trend that HI subjects performed slightly better than NH subjects, although the difference was not significant. CI subjects also showed quite good performance on temporal modulation detection at 10 Hz, consistent with Won et al. (2011) [26]. Given the fact that both the spectral and temporal modulation detection tests used a relatively slow modulation frequency in the current study, it is interesting to note that there was a marked difference in spectral modulation detection performance between electric hearing (i.e., CI subjects) and acoustic hearing (i.e., NH and HI subjects), but for temporal modulation detection, difference in performance was relatively small. We speculate that, for slow modulation frequencies, the effect of the CI-auditory nerve interface may be greater on spectral processing than on temporal processing. This speculation is partly consistent with the previous findings that the degree of spread of excitations due to channel interactions in CIs is significantly correlated with spectral resolution measured by spectral-ripple discrimination [37] but not with temporal modulation detection at 50 Hz [36].

### C. Contribution of spectral and temporal modulation upon speech perception

In the current study, significant correlations were found between STM detection thresholds and sentence recognition in quiet and noise across the three subject groups (Fig 7). Visual inspection on Fig 7A reveals that there was a plateau in K-CID sentence recognition in quiet (~100%) when STM detection thresholds ranged between -15 and -25 dB. There was little variability in K-CID sentence recognition scores across NH and HI subjects, but CI subjects showed a wide range of performance between 0 and 100%. In contrast, a substantial variability was observed in both HI and CI subjects for HINT sentence recognition in noise. There was also a plateau in HINT sentence recognition in noise when STM detection thresholds ranged between -15 and -20 dB. For both sentence recognition in quiet and in noise, subjects with less than -15 dB STM detection thresholds tended to show very good sentence identification performance.

With regard to CI subjects, there were significant correlations between STM detection performance and speech identification performance (Fig 8). The strength of correlations, however, differed slightly across the STM stimulus conditions. Importantly, there was a pattern that STM stimuli with lower spectral densities (0.5 and 1.0 c/o) tended to show significant correlations with speech identification performance, largely consistent with previous reports [11, 12, 33]. That is, Saoji et al. (2009) [33] and Anderson et al. (2012) [12] demonstrated that spectral modulation detection thresholds at lower spectral densities (0.25 to 0.5 c/o) showed stronger correlations for speech perception abilities in CI users, but correlations between speech perception abilities and spectral modulation detection thresholds at higher spectral densities (1.0 to 3.0 c/o) were less robust. Using 22 bimodal listeners with a CI in one ear and low-frequency acoustic hearing in the non-implanted ear, Zhang et al. (2013) [11] showed a significant correlation between spectral modulation detection thresholds at 1 c/o and the benefit for speech perception when the acoustic and electric stimulation were combined compared to the CI alone. As argued by Saoji et al. [33] and Anderson et al. [12], it may be possible that speech perception for CI users might not require to process high frequency spectral modulation cues, but the sensitivity to slow spectral modulation patterns may play the primary role.

In order to elucidate the relative contribution of spectral and temporal modulation cues upon speech perception abilities in CI users, partial correlations were performed controlling for the contribution of either spectral or temporal modulation detection thresholds (Table 5). When the effect of spectral modulation detection at 1 c/o was factored out, correlations between STM detection performance and sentence identification performance became no longer significant. In contrast, factoring out the effect of temporal modulation detection at 10 Hz did not affect the correlations between STM detection and sentence recognition. The effect of temporal modulation detection might have been small because the correlation between temporal modulation detection at 10 Hz and sentence recognition was weaker (r = -0.33, *p* = 0.015 for K-CID in quiet; *r* = -0.23, p = 0.13 for HINT in noise). Previously, Won et al. (2011b) evaluated the relationship between speech perception in quiet and noise and temporal modulation detection as a function of modulation frequency. In Won et al., significant correlations were found for relatively higher modulation frequencies (75–300 Hz), but not for relatively slow temporal modulation frequencies (10 and 50 Hz). Similarly, Gnansia et al. (2013) [24] also showed that temporal modulation detection abilities for slow modulation frequency (8 Hz) were not always predictive of speech perception abilities in noise for CI users. Interestingly, as noted above, CI users appear to utilize slow spectral modulation patterns for speech perception both in quiet and noise. Taken together, these results suggest that CI users may put more emphasis on slow spectral modulation cues rather than slow temporal modulation cues for speech perception.

### D. Implications for cochlear implant research

The current study demonstrated that STM detection may be a potentially useful measure of performance for CI users. First, a wide range of performance was observed across three different subject groups, suggesting that the STM detection test is sensitive to altered encoding of STM cues due to the CI-auditory nerve interface. Second, the results from CI users also showed a broad range, demonstrating potential utility to evaluate a wide range of CI performance. Third, CI user’s STM modulation detection performance for low spectral densities was significantly correlated with sentence recognition in quiet and in noise. Fourth, test–retest analysis revealed that the STM detection test was reliable and does not show a significant learning effect. Altogether, the results of the current study demonstrate that low spectral density STM detection may be a viable diagnostic and research tool for evaluating speech perception capabilities in CI users.

As noted in the introduction, STM stimuli in the current study are similar to Schroeder-phase stimuli [13, 44] in that the broadband acoustic FM patterns are encoded via the so-called “FM-to-AM conversion” process through the sound processor. The FM-to-AM conversion occurs for CI electrode outputs when the differential attenuation of CI sound processor’s digital filters produces the conversion of the frequency excursions of FM into the dynamic variations of the output levels of the filters. The extent to which the FM-to-AM conversion occurs depends on the frequency selectivity of the auditory system. For example, Lorenzi et al. (2012) [45] showed that the broadening of cochlear filters associated with sensorineural hearing loss reduces the HI listeners’ ability to identify speech based on the AM cues recovered from the broadband FM speech signals, when cochlear filters are broadened by a factor greater than two. Likewise, if CI users are programmed with a sixteen-channel sound coding strategies, the bandwidth of the digital filters is approximately two times wider than the bandwidth of the normal auditory filters. Nevertheless, recent studies [37, 46] have demonstrated that CI users can make efficient use of AM cues recovered from speech FM cues both in quiet and in challenging listening environments, despite poor frequency selectivity. Therefore, significant correlations found in the current study between STM detection thresholds and sentence identification performance provide a further evidence that the sensitivity to AM cues recovered from broadband FM signals may be an important factor contributing to speech perception capabilities for CI users.

This point is timely important because CI manufacturers and investigators are making efforts to better represent the acoustic FM information through biphasic pulsatile stimulation strategies. Won et al. (2014) [46] demonstrated that the ability of CI users to use recovered AM cues from broadband FM speech cues plays an important role in speech perception for acoustic environments where original speech cues are severely distorted. In this regard, it is noteworthy that a measure of sentence identification in steady-state background noise was used in the current study. However, CI users receive STM information of the target speech that is degraded by multiple sources such as fluctuating background noise such as competing speech signals and environmental sounds, and reverberation. Furthermore, STM speech cues are delivered to CI users in a distorted fashion because of various sound processor settings (e.g., front-end sound processing such as noise reduction schemes, beamforming, and sound processing map settings such as input dynamic range, number of channels, frequency-to-electrode allocation, and etc.). Therefore, it will be important to evaluate STM detection performance for different sound processor settings and assess the relationship to speech perception capabilities when target speech is degraded by multiple sources existing in daily listening situations. Finally, future studies should also investigate STM detection performance for different stimulation modes, including electro-acoustic stimulation, bimodal and bilateral stimulation. For these purposes, the STM detection test could serve as an efficient, non-linguistic tool to estimate CI users’ sensitivity to use recovered AM cues from broadband FM signals.

### Summary

The current study showed the following:

1. A wide range of STM detection performance was observed across NH, HI, and CI subjects, indicating the potential influence of altered encoding of STM cues for HI and CI listeners.
2. High levels of STM detection performance were observed in some CI users in comparison to NH and HI subjects where STM cues were transmitted solely based on AM cues recovered from broadband FM cues.
3. Test-retest reliability for the STM detection test was good, and no learning was observed.
4. Significant correlations were found between STM detection thresholds for low spectral densities and sentence identification in quiet and in noise.
5. Partial correlation analyses controlling for the effects of either spectral or temporal modulation detection suggest that slow spectral modulation rather than slow temporal modulation may be important for determining speech perception capabilities for CI users.

## Supporting Information

S1 Dataset Raw data file of each group. (XLSX)

## References

[1] Wilson BS, Dorman MF. Cochlear implants: a remarkable past and a brilliant future. Hear Res. 2008;242: 3–21. 10.1016/j.heares.2008.06.005 18616994 PMC3707130

[2] Zeng FG, Rebscher S, Harrison W, Sun X, Feng H. Cochlear implants: system design, integration, and evaluation. IEEE Rev Biomed Eng. 2008;1: 115–142. 10.1109/RBME.2008.2008250 19946565 PMC2782849

[3] Anderson ES, Nelson DA, Kreft H, Nelson PB, Oxenham AJ. Comparing spatial tuning curves, spectral ripple resolution, and speech perception in cochlear implant users. J Acoust Soc Am. 2011;130: 364–375. 10.1121/1.3589255 21786905 PMC3155592

[4] Henry BA, Turner CW. The resolution of complex spectral patterns by cochlear implant and normal-hearing listeners. J Acoust Soc Am. 2003;113: 2861–2873. 12765402 10.1121/1.1561900

[5] Henry BA, Turner CW, Behrens A. Spectral peak resolution and speech recognition in quiet: normal hearing, hearing impaired, and cochlear implant listeners. J Acoust Soc Am. 2005;118: 1111–1121. 16158665 10.1121/1.1944567

[6] Supin A, Popov VV, Milekhina ON, Tarakanov MB. Frequency resolving power measured by rippled noise. Hear Res. 1994;78: 31–40. 7961175 10.1016/0378-5955(94)90041-8

[7] Won JH, Drennan WR, Rubinstein JT. Spectral-ripple resolution correlates with speech reception in noise in cochlear implant users. J Assoc Res Otolaryngol. 2007;8: 384–392. 17587137 10.1007/s10162-007-0085-8 PMC2538435

[8] Won JH, Jones GL, Drennan WR, Jameyson EM, Rubinstein JT. Evidence of across-channel processing for spectral-ripple discrimination in cochlear implant listeners. J Acoust Soc Am. 2011;130: 2088–2097. 10.1121/1.3624820 21973363 PMC3206911

[9] Eddins DA, Bero EM. Spectral modulation detection as a function of modulation frequency, carrier bandwidth, and carrier frequency region. J Acoust Soc Am. 2007;121: 363–372. 17297791 10.1121/1.2382347

[10] Litvak LM, Spahr AJ, Saoji AA, Fridman GY. Relationship between perception of spectral ripple and speech recognition in cochlear implant and vocoder listeners. J Acoust Soc Am. 2007;122: 982–991. 17672646 10.1121/1.2749413

[11] Zhang T, Spahr AJ, Dorman MF, Saoji A. Relationship between auditory function of nonimplanted ears and bimodal benefit. Ear Hear. 2013;34: 133–141. 23075632 10.1097/AUD.0b013e31826709af PMC3549325

[12] Anderson ES, Oxenham AJ, Nelson PB, Nelson DA. Assessing the role of spectral and intensity cues in spectral ripple detection and discrimination in cochlear-implant users. J Acoust Soc Am. 2012;132: 3925–3934. 10.1121/1.4763999 23231122 PMC3529540

[13] Drennan WR, Won JH, Nie K, Jameyson E, Rubinstein JT. Sensitivity of psychophysical measures to signal processor modifications in cochlear implant users. Hear Res. 2010;262: 1–8. 10.1016/j.heares.2010.02.003 20144699 PMC2864608

[14] Middlebrooks JC. Cochlear-implant high pulse rate and narrow electrode configuration impair transmission of temporal information to the auditory cortex. J Neurophysiol. 2008;100: 92–107. 10.1152/jn.01114.2007 18450583 PMC2493502

[15] Won JH, Nie K, Drennan WR, Rubinstein JT. Maximizing the spectral and temporal benefits of two clinically used sound processing strategies for cochlear implants. Trends Amplif. 2012;16: 201–210. 10.1177/1084713812467855 23264570 PMC3531870

[16] Fu QJ. Temporal processing and speech recognition in cochlear implant users. Neuroreport. 2002;13: 1635–1639. 12352617 10.1097/00001756-200209160-00013

[17] Kong YY, Deeks JM, Axon PR, Carlyon RP. Limits of temporal pitch in cochlear implants. J Acoust Soc Am. 2009;125: 1649–1657. 10.1121/1.3068457 19275322

[18] Shannon RV. Temporal modulation transfer functions in patients with cochlear implants. J Acoust Soc Am. 1992;91: 2156–2164. 1597606 10.1121/1.403807

[19] Townshend B, Cotter N, Van Compernolle D, White RL. Pitch perception by cochlear implant subjects. J Acoust Soc Am. 1987;82: 106–115. 3624633 10.1121/1.395554

[20] Garadat SN, Zwolan TA, Pfingst BE. Across-site patterns of modulation detection: relation to speech recognition. J Acoust Soc Am. 2012;131: 4030–4041. 10.1121/1.3701879 22559376 PMC3356319

[21] Pfingst BE, Bowling SA, Colesa DJ, Garadat SN, Raphael Y, Shibata SB, et al Cochlear infrastructure for electrical hearing. Hear Res. 2011;281: 65–73. 10.1016/j.heares.2011.05.002 21605648 PMC3208788

[22] Bacon SP, Viemeister NF. Temporal modulation transfer functions in normal-hearing and hearing-impaired listeners. Audiology. 1985;24: 117–134. 3994589 10.3109/00206098509081545

[23] Cazals Y, Pelizzone M, Saudan O, Boex C. Low-pass filtering in amplitude modulation detection associated with vowel and consonant identification in subjects with cochlear implants. J Acoust Soc Am. 1994;96: 2048–2054. 7963020 10.1121/1.410146

[24] Gnansia D, Lazard DS, Leger AC, Fugain C, Lancelin D, Meyer B, et al Role of slow temporal modulations in speech identification for cochlear implant users. Int J Audiol. 2014;53: 48–54. 10.3109/14992027.2013.844367 24195655

[25] Viemeister NF. Temporal modulation transfer functions based upon modulation thresholds. J Acoust Soc Am. 1979;66: 1364–1380. 500975 10.1121/1.383531

[26] Won JH, Drennan WR, Nie K, Jameyson EM, Rubinstein JT. Acoustic temporal modulation detection and speech perception in cochlear implant listeners. J Acoust Soc Am. 2011;130: 376–388. 10.1121/1.3592521 21786906 PMC3155593

[27] Chi T, Gao Y, Guyton MC, Ru P, Shamma S. Spectro-temporal modulation transfer functions and speech intelligibility. J Acoust Soc Am. 1999;106: 2719–2732. 10573888 10.1121/1.428100

[28] Elhilali M, Chi T, Shamma SA. A spectro-temporal modulation index (STMI) for assessment of speech intelligibility. Speech Commun. 2003;41: 331–348.

[29] Bernstein JG, Mehraei G, Shamma S, Gallun FJ, Theodoroff SM, Leek MR. Spectrotemporal modulation sensitivity as a predictor of speech intelligibility for hearing-impaired listeners. J Am Acad Audiol. 2013;24: 293–306. 10.3766/jaaa.24.4.5 23636210 PMC3973426

[30] Mehraei G, Gallun FJ, Leek MR, Bernstein JG. Spectrotemporal modulation sensitivity for hearing-impaired listeners: dependence on carrier center frequency and the relationship to speech intelligibility. J Acoust Soc Am. 2014;136: 301–316. 10.1121/1.4881918 24993215 PMC4187385

[31] Jang JH, Chang HK, Park HY, Yoo JC, An YH, Lee JH, et al Comparison Analysis between Korean Central Institute for the Deaf Sentence and Korean Hearing in Noise Test Sentence. Korean J Otorhinolaryngol-Head Neck Surg. 2012;55: 85–89.

[32] Moon SK, Hee Kim S, Ah Mun H, Jung HK, Lee JH, Choung YH, et al The Korean hearing in noise test. Int J Audiol. 2008;47: 375–376. 10.1080/14992020701882457 18569115

[33] Saoji AA, Litvak L, Spahr AJ, Eddins DA. Spectral modulation detection and vowel and consonant identifications in cochlear implant listeners. J Acoust Soc Am. 2009;126: 955–958. 10.1121/1.3179670 19739707

[34] Friesen LM, Shannon RV, Baskent D, Wang X. (2001) Speech recognition in noise as a function of the number of spectral channels: comparison of acoustic hearing and cochlear implants. J Acoust Soc Am. 110(2):1150–63. 11519582 10.1121/1.1381538

[35] Başkent D. (2006) Speech recognition in normal hearing and sensorineural hearing loss as a function of the number of spectral channels. J Acoust Soc Am. 120:2908–25. 17139748 10.1121/1.2354017

[36] Jones GL, Won JH, Drennan WR, Rubinstein JT. Relationship between channel interaction and spectral-ripple discrimination in cochlear implant users. J Acoust Soc Am. 2013;133: 425–433. 10.1121/1.4768881 23297914 PMC3548834

[37] Won JH, Humphrey EL, Yeager KR, Martinez AA, Robinson CH, Mills KE, et al Relationship among the physiologic channel interactions, spectral-ripple discrimination, and vowel identification in cochlear implant users. J Acoust Soc Am. 2014;136: 2714–2725. 10.1121/1.4895702 25373971

[38] Levitt H. Transformed up-down methods in psychoacoustics. J Acoust Soc Am. 1971;49: 467 5541744

[39] Moore BC, Glasberg BR. Temporal modulation transfer functions obtained using sinusoidal carriers with normally hearing and hearing-impaired listeners. J Acoust Soc Am. 2001;110: 1067–1073. 11519575 10.1121/1.1385177

[40] Benjamini Y., Hochberg Y. (1995) “Controlling the false discovery rate—A practical and powerful approach to multiple testing,” J. R. Stat. Soc. B. Met. 57, 289–300.

[41] Hughes M. L., Stille L. J. (2010) “Effect of stimulus and recording parameters on spatial spread of excitation and masking patterns obtained with the electrically evoked compound action potential in cochlear implants,” Ear. Hear. 31, 679–692. 20505513 10.1097/AUD.0b013e3181e1d19e PMC2932804

[42] Jeon EK, Turner CW, Karsten SA, Gantz BJ, Henry BA (2015) Comparison of spectral ripple noise resolution obtaind from more recently implanted CI users and previously published data Presented at the 2015 Conference on Implantable Auditory Prostheses. Lake Tahoe, CA Abstract book page 224.

[43] Füllgrabe C., Meyer B., Lorenzi C. (2003) Effect of cochlear damage on the detection of complex temporal envelopes. Hear. Res., 178, 35–43. 12684175 10.1016/s0378-5955(03)00027-3

[44] Imennov NS, Won JH, Drennan WR, Jameyson E, Rubinstein JT. Detection of acoustic temporal fine structure by cochlear implant listeners: behavioral results and computational modeling. Hear Res. 2013;298: 60–72. 10.1016/j.heares.2013.01.004 23333260 PMC3605703

[45] Lorenzi C, Wallaert N, Gnansia D, Leger AC, Ives DT, Chays A, et al Temporal-envelope reconstruction for hearing-impaired listeners. J Assoc Res Otolaryngol. 2012;13: 853–865. 10.1007/s10162-012-0350-3 23007719 PMC3505588

[46] Won JH, Shim HJ, Lorenzi C, Rubinstein JT. (2014) Use of amplitude modulation cues recovered from frequency modulation for cochlear implant users when original speech cues are severely degraded. J Assoc Res Otolaryngol.15(3):423–39 10.1007/s10162-014-0444-1 24532186 PMC4010597

